# Neurologists’ current practice and perspectives on communicating the diagnosis of a motor neurodegenerative condition: a UK survey

**DOI:** 10.1186/s12883-021-02062-6

**Published:** 2021-01-22

**Authors:** Eleftherios Anestis, Fiona J. R. Eccles, Ian Fletcher, Jane Simpson

**Affiliations:** Division of Health Research, Faculty of Health and Medicine, Lancaster University, Lancaster, LA1 4YT UK

**Keywords:** Breaking bad news, Diagnosis communication, Patient-provider communication, Neurodegenerative, Motor neurone disease, Multiple sclerosis, Parkinson’s disease, Huntington’s disease

## Abstract

**Background:**

The communication of a life-changing diagnosis can be a difficult task for doctors with potential long-term effects on patient outcomes. Although several studies have addressed the experiences of individuals with motor neurodegenerative diseases in receiving this diagnosis, a significant research gap exists regarding professionals’ perspectives, especially in the UK. This study aimed to assess UK neurologists’ current practice and perspectives on delivering the diagnosis of a motor neurodegenerative disease, explore different aspects of the process and detail the potential challenges professionals might face.

**Methods:**

We conducted an anonymised online survey with 44 questions, grouped into four sections; basic demographic information, current practice, the experience of breaking bad news and education and training needs.

**Results:**

Forty-nine professionals completed the survey. Overall, participants seemed to meet the setting-related standards of good practice; however, they also acknowledged the difficulty of this aspect of their clinical work, with about half of participants (46.5%) reporting moderate levels of stress while breaking bad news. Patients’ relatives were not always included in diagnostic consultations and participants were more reluctant to promote a sense of optimism to patients with poorer prognosis. Although professionals reported spending a mean of around 30–40 min for the communication of these diagnoses, a significant proportion of participants (21–39%) reported significantly shorter consultation times, highlighting organisational issues related to lack of capacity. Finally, the majority of participants (75.5%) reported not following any specific guidelines or protocols but indicated their interest in receiving further training in breaking bad news (78.5%).

**Conclusions:**

This was the first UK survey to address neurologists’ practice and experiences in communicating these diagnoses. Although meeting basic standards of good practice was reported by most professionals, we identified several areas of improvement. These included spending enough time to deliver the diagnosis appropriately, including patients’ relatives as a standard, promoting a sense of hope and responding to professionals’ training needs regarding breaking bad news.

**Supplementary Information:**

The online version contains supplementary material available at 10.1186/s12883-021-02062-6.

## Background

Breaking bad news is a critical and distressing process for patients but also an often stressful and challenging task for clinicians [1, 2] Bad news in medicine refers to ‘any information likely to alter drastically a patient’s view of his or her future’ (p. 1597) [3] such as the communication of the diagnosis of a potentially life-changing condition. How a diagnosis is delivered can have a long-term impact on patient outcomes such as treatment adherence [1], psychological adjustment and involvement in treatment decision making [4], understanding of the condition [5] and satisfaction with care [6]. From the doctor’s perspective, breaking bad news can be an emotionally burdensome and intrinsically difficult task, with factors such as time constraints, intercultural differences in relation to diagnosis disclosure and lack of private space making it even more challenging [7].

Most studies on the delivery of bad news have been conducted within the field of oncology. However, the delivery of bad news can be a critical issue in other medical specialties such as neurology. Storstein [8] argues that when breaking bad news, neurologists deal with specific challenges that relate to particular medical considerations and the emotional aspects of neurological diseases. In particular, several chronic neurological conditions, such as Parkinson’s disease (PD), multiple sclerosis (MS) and Huntington’s disease (HD), are incurable, have a progressive nature and impact both physical and cognitive functions [8], while others, such as motor neurone disease (MND), can also be more immediately life threatening [9]. A scoping review of doctors’ and patients’ perspectives on giving and receiving the diagnosis of MND, MS or PD [10] revealed mixed results regarding patients’ experiences and satisfaction with how diagnosis delivery was handled. The main factors which contributed to negative patient experiences were the often-limited duration of the consultation, inadequate information provision and a perceived insensitive approach by the professional breaking the news. Moreover, the review found a significant research gap on studies addressing the physicians’ perspectives, which could offer a better understanding of the doctor-patient interactions at the time of the diagnosis.

The aim of this study was to assess UK neurologists’ current practice when delivering the diagnosis of a motor neurodegenerative disease (MNDD), in particular PD, MS, HD and MND. Currently, there are no UK studies on this topic, the aim of the study was to explore different aspects of the process, such as the setting, duration and challenges of communicating a diagnosis of this nature. In addition, potential factors affecting practice and differences between delivering the diagnosis for different conditions were also explored. As the results are descriptive, no hypotheses were made.

## Method

The study was approved by both the authors’ host institution’s research ethics committee and the Health Research Authority, a unified system for the governance of health research in the UK.

The questionnaire used for this study was constructed after a comprehensive review of the relevant literature on breaking bad news and guidelines such as SPIKES, the Six-Step Protocol for Delivering Bad News [11] and the National Institute for Health and Care Excellence (NICE) guidelines for the management of MND [12], MS [13] and PD [14]. It was also largely based on the questionnaire used by Aoun et al. [15] for a similar study on neurologists’ experiences on delivering the diagnosis of MND in Australia. The first draft of the survey was reviewed by two practising neurologists for clarity and relevance and adjustments were made based on their comments.

The survey was hosted online on the Qualtrics platform and was open for 2 years (from September 2018 to September 2020). Eligible participants were medical professionals, including specialist registrars, practising in the UK who had experience in delivering the diagnosis for at least one of the conditions included in the survey. The survey comprised 44 questions grouped into four sections; demographic information, current practice, the experience of breaking bad news and education and training needs (Additional File 1). It was completed anonymously, and questions were mainly closed with several open-ended questions where participants were asked to elaborate on their answers or provide any further comments. Participants were recruited through the Association of British Neurologists (ABN), other associations related to neurology or MNDDs and through collaborations with National Health Service (NHS) trusts.

Data from the closed questions were imported and analysed in IBM SPSS 26 software package [16], using descriptive statistics; means, standard deviations, range and frequencies. In addition, qualitative data from the open-ended questions of the survey were used to enhance, explain and expand the findings from the analysis of the quantitative data. Respondents who completed less than 50% of the survey (*N* < 5) were excluded from the study.

## Results

### Participants profile

Forty-nine professionals responded to the survey; 43 consultant neurologists, 4 neurology specialist registrars, one consultant neuropsychiatrist and one clinical fellow. Participants were mainly male (67%), almost half of them were in the 41–50 age group (48%) and had a mean of 10 years of experience (ranging from less than one to 23: *SD* = 6.8). Almost all participants mainly practised in England, 5 participants mainly practised in Wales and although all participants practised in the NHS, 12 participants were also practising privately. See Table 1 for a summary of participants’ demographics.

**Table 1:** Participants’ characteristics

Participants’ role:	Number of participants (%)
Consultant neurologist	43 (88%)
Neurology specialist registrar	4 (8%)
Consultant neuropsychiatrist	1 (2%)
Clinical fellow	1 (2%)
**Gender ***(one response missing)*	
Male	32 (67%)
Female	16 (33%)
**Age ***(one response missing)*	
31–40	13 (27%)
41–50	23 (48%)
51–60	11 (23%)
61 or older	1 (2%)
**Experience in delivering the diagnosis**	
Parkinson’s disease	44 (90%)
Multiple sclerosis	41 (84%)
Motor neurone disease	43 (88%)
Huntington’s disease	33 (67%)

### Diagnosis disclosure

Most participants had experience in communicating all four diagnoses under review; 90% of professionals had experience in breaking bad news for PD, 88% for MND, 84% for MS and 67% for HD. Most of the professionals who had experience in delivering the diagnosis of HD (73%) had only communicated 1–20 diagnoses, which can be explained by the rarity of the condition and the diagnosis of onset^1^ of HD potentially being given mostly in specialist clinics.

The vast majority of professionals (87%) reported always disclosing the diagnosis for these conditions to the patients. Text comments highlighted that it would be ‘fundamentally unethical’ not to inform a patient of their diagnosis. Participants believed that being honest and transparent about the diagnosis helped with the management of the condition and building a relationship with the patient. However, some comments indicated that professionals would not disclose the diagnosis only when patients had clearly stated that they did not wish to know or when the diagnosis was not definite and further investigation was required. Moreover, 30% of participants reported that they would sometimes refer patients to other medical professionals who would then deliver the diagnosis. Qualitative comments indicated that they would follow this approach when they were uncertain about a diagnosis or they could refer to a specialist clinic.

### Setting, time and people involved in the consultation

When asked about the setting of the consultation, 74% of participants reported ‘always’ delivering the diagnosis in a private space and 96% stated that ‘most of the time’ or ‘always’ the diagnosis was communicated without any interruptions. In addition, 75% of professionals reported always maintaining eye contact with the patient and 75% arranged to have suitable seating at the same level as the patient without a desk or barrier.

On average, professionals reported investing around 30 min for the delivery of the diagnosis for PD (*M* = 30, *SD* = 9.3), MS (*M* = 28.7, *SD* = 10.4) and HD (*M* = 29.9, *SD* = 16.5) and 41 min (*SD* = 26) for MND. However, a considerable proportion of participants (21% for PD, 32% for MS, 39% for HD and 20% for MND) reported spending 15 to 20 min for the diagnosis consultation and 30% of participants reported spending over an hour to communicate the diagnosis of MND. More than half of professionals (64%) believed patients were given enough time to ask questions and express their emotions. However, across conditions, 58–69% of professionals ‘sometimes’ needed more than one consultation to explain these diagnoses and 23–35% ‘always’ needed more consultations. One participant explained that diagnosis communication was a more dynamic process, beyond the diagnostic consultation:

*‘I do not think that breaking the diagnosis is really a one-off event (even if you had all the time in the world), but rather a process that continues throughout much of the time that you look after an individual as the disease and the patient’s relationship with it often change as time goes on.’*

Furthermore, 72% of professionals did not refrain from giving a diagnosis at any specific time or day, and those who did so explained that they avoided giving bad news at a late appointment or before the weekend if the patient was not accompanied by someone and also before patients’ birthdays or before holidays such as Christmas.

Regarding the involvement of other people in the consultation, 60% of professionals stated that ‘most of the time’ or ‘always’, patients were asked to bring someone to the consultation, however 15% reported that patients were not asked to bring someone. In addition, 53% of participants ‘sometimes’ included other healthcare professionals in the consultation and 19% ‘never’ did so.

### Content of the consultation / information giving

Almost all participants agreed that how the diagnosis was reached (96%), treatment options (96%), the degree of certainty of the diagnosis (92%) and the course/prognosis of the disease (90%) were topics that should be discussed with the patient at diagnosis. Causes of the disease (76%) and current research (63%) were also considered important topics to be covered. Additional comments showed that neurologists also chose to discuss other important topics, such as the family, hereditary and legal implications of the diagnosis (e.g. driving), information on the support plan and other healthcare professionals who would be involved in their care and signposting to related charities and reputable sources of information. In addition to oral information, 28% of professionals ‘always’ provided patient-tailored information in written form and 43% did so ‘most of the time’. Information on local support groups and national charities was ‘always’ shared by about half of the participants for PD, MS and HD and by 67% of participants for MND. When asked whether they promoted a feeling of optimism when delivering a diagnosis, more respondents reported ‘probably’ or ‘definitely’ promoting hope in PD (91%) and MS (90%) than HD (39%) and MND (31%).

### Personal experiences and challenges in breaking bad news for MNDDs

Regarding the perceived difficulty of diagnosis communication for these conditions, 54% of participants believed that it was ‘definitely’ and 23% that it was ‘probably’ a difficult task for the physician. Most professionals (74%) agreed that being honest without taking away hope was the most challenging part of communicating the diagnosis of MNDDs, followed by spending the right amount of time (47%). Dealing with the patient’s emotional reaction (25%), involving the family of the patient (14%) and involving the patient or family in decision making (12%) were considered difficult by fewer participants. When asked about how often they faced several potential barriers during a breaking bad news consultation, professionals reported that fear of causing distress (32.5%), excessive workload (32.5%) and perceived lack of time (30%), were among the most often experienced barriers, which they faced ‘most of the time’ or ‘always’. Conversely, fear of the ‘messenger getting blamed for bad news’ and lack or insufficient training in breaking bad news were not often experienced as barriers. In addition, 46.5% of respondents reported experiencing moderate and 9% reported high to very high levels feelings of stress and anxiety during the delivery of these diagnoses, while only 12% reported not experiencing such feelings at all.

Overall, most professionals (61%) believed they were ‘good’ at communicating the diagnosis of a MNDD, 23% assessed themselves as ‘very good’ and none thought they were ‘poor’ at it. For PD and MS, more than half of the respondents were confident to very confident (63%) that patients left the consultation having taken in all the information relevant to them at that point. However, for the case of HD and MND, 61 and 58% of professionals respectively were ‘not sure’ to ‘really not confident’ that patients had taken in all the relevant information. In general, 81% believed patients were ‘somewhat satisfied’ to ‘very satisfied’ with how the diagnosis was delivered.

### Strategies and training on breaking bad news

In the last part of the survey, participants were asked to report on the strategies they employed and the training they had received in breaking bad news. Most professionals (75.5%) reported not following any specific strategy or best practice guidelines when delivering an MNDD diagnosis. Those who did explained that they followed NICE guidelines and were familiar with research on best practice and breaking bad news. Most professionals (83%) had received some kind of training on breaking bad news, either as a part of their formal education, clinical training or by sitting in with other clinicians who broke bad news. Qualitative comments also showed that respondents had learnt how to break bad news through experience, advanced communication skills training and generic training on breaking bad news, although the latter had focused on cancer. Around 31% had received no training in techniques of responding to patients’ emotions and, for those who had, they reported having received such training as a part of their degree or developed these skills through experience and observing others breaking bad news. Finally, most participants (78.5%) were somewhat to very interested in receiving further education on breaking bad news and on techniques for how to respond best to patients’ emotional needs.

### Qualitative comments

Most qualitative comments given by participants were related to the challenges of communicating the diagnosis of an MNDD. Two common issues for professionals were related to limited consultation times and the lack of capacity to schedule a follow-up with the patient soon after diagnosis with some follow-up appointments booked for even 15 months post-diagnosis. Therefore, especially in general neurology clinics, participants had to cover many different topics in one single consultation, although the official time slot allocated for the appointment was not long enough:

‘*Given current waiting lists for some of my movement disorders clinics, it may be 9 months before I next see a newly diagnosed PD patient. I therefore not only have to explain the diagnosis, pathogenesis, and treatment options but also explain the treatment plan and contingencies for possible hiccups to cover a ridiculously large period of time in (officially) fifteen minutes. Is it any surprise my clinics (overruns) by several hours.*’

‘*Insufficient time for vast amount of information to be usefully imparted. Pregnancy discussions alone merit a full consultation*.’

Conversely, a participant who was also practising privately reported that they could ‘*see patients again within a week to go over questions and discuss treatment plans once dust has settled’*.

Several professionals talked about this lack of capacity as ‘*a service delivery issue*’, which, apart from limited consultation time, involved insufficient access to nurses and administrative staff who could coordinate these appointments: ‘*Someone (is needed) to coordinate* (the) *pathway so everything* (is) *available at consultation: relative, nurse, info etc.’*. Specialist clinics seemed to be able to offer a better service, however one participant commented that referrals were not always possible when there were no specialist services locally.

Apart from organisational factors which affected their practice, professionals addressed how various illness and patient-related factors could affect their diagnostic practice. Diagnostic and prognostic uncertainty were common issues for participants delivering MNDD diagnoses. One person highlighted feeling ‘pressured’ by patients to give a diagnosis, even though they had not reached diagnostic certainty. Similarly, it was not always possible to share prognostic information, for example regarding the rate of progression and the potential level of future impairment. In addition, it was often commented that the lack of curative treatments made breaking bad news more difficult, especially when patients were initially unaware of the incurable nature of their condition. However, being able to offer symptom management for PD and disease modifying treatments for MS made the process of diagnosis delivery more positive.

On an emotional level, professionals reported several patient-related factors that made breaking bad news more challenging:

‘*At times a patient’s situation particularly resonates and this can be emotionally draining on the clinician.*’

Participants mentioned several cases that were particularly challenging, such as delivering a PD diagnosis to young people, delivering an MS diagnosis to young women who wanted to have children (‘*shattering hopes*’), delivering an HD diagnosis to people with children or delivering the diagnosis of MND to a patient who was presenting rapid progression or with already advanced symptoms at diagnosis. One professional used the word ‘*despondency*’ to describe how they felt when delivering such diagnoses.

## Discussion

This is the first UK survey study to address doctors’ practice and experiences in communicating the diagnosis of an MNDD.

Generally, participants seemed to meet the setting-related standards of good practice [11] in breaking bad news by communicating the diagnosis in a private space, avoiding interruptions, arranging suitable seating and maintaining eye contact with patients. Regarding involving other people in the consultation, there was room for improvement since only 21.3% of professionals always asked patients to bring someone in consultation, 38.3% did so most of the time and 15% never did. One participant highlighted the fact that asking a patient to bring someone with them might act as a warning and could also increase their distress prior to the consultation and affect how much information they could absorb. However, although involving patients’ relatives in a diagnostic consultation can be a challenge for healthcare professionals, they can offer emotional support, serve as the patient’s advocate and receive important information they will need if they act as the patient’s primary caregivers [17, 18]. Moreover, several MNDDs guidelines specifically recommend or imply that, with the patient’s agreement, their support network should be present at diagnosis [12–14, 19].

Consultation duration reported by professionals in this survey was not always optimal and qualitative comments showed that organisational factors affected how much time they could invested for diagnostic consultations. Participants reported spending a mean of around 30 min to deliver the diagnosis of PD, MS and HD and 41 min for MND, however there was a considerable percentage of professionals (20–39%) who reported spending 15–20 min. The latter falls short compared to the European Federation of Neurological Societies (EFNS) recommended guideline of 45 to 60 min for the diagnosis of MND [19], however there are no published guidelines on consultation times for the other MNDDs. These findings correspond with both UK [20–22] and international [23–27] MNDD patient studies which have reported short consultation times that often led to patient dissatisfaction. Even though participants in this study reported sharing information on how the diagnosis was reached, the impact of the condition on patients’ lives and their care plan, they still believed patients left the consultation not having taken in all information relevant to them at the point of diagnosis, especially for the case of MND and HD. This is possibly linked to limited consultation times or, as one participant noted, due to patients’ state of shock which affects how much information they can absorb. Professionals reported that they would often need more than one consultation to fully deliver the diagnosis. However, this is challenging since, particularly for PD, qualitative comments highlighted issues related to lack of capacity to book early follow-ups with some consultations being booked even 15 months post-diagnosis.

Most professionals agreed that diagnosis communication for MNDDs was a difficult task and being honest without taking away hope was the most challenging aspect of the consultation, a challenge which has also been reported by Aoun’s survey of neurologists in Australia [15] and professionals working in other medical specialties such as oncology [7]. Participants in this study reported being particularly reluctant to promote a feeling of optimism when delivering the diagnosis of HD and MND. As some qualitative comments suggest, this could be associated with the poor prognosis for these conditions, however EFNS guidelines for MND [19] encourage professionals to discuss reasons for hope, such as ongoing research, drug trials and the variability of the disease and specifically advise against not providing hope during diagnosis. It should also be noted that providing hope is not always analogous to indicating the possibility of a cure. Hope can be generated for the optimal management of the condition, in whatever form that has to take. Instilling hope therefore can take many forms and is an important aspect of the patient’s rehabilitation [28]. Feelings of hopelessness in people with MND have been reported to be more strongly correlated to quality of life than their physical functioning [29] and dissatisfaction with information delivery can negatively influence patient’s sense of hope [30]. Moreover, a review by Clayton et al. [31] showed that although most patients approaching end of life prefer honest and accurate information, they are also able to maintain a sense of hope. The review suggested that healthcare professionals should recognise and foster different and realistic forms of hope relevant to the particular patient and their family by carefully assessing patients’ information preferences and emphasising on what can be done for them.

Participants in this survey were also asked about the emotional aspects of delivering the diagnoses of MNDDs. More than half of professionals reported that they experienced moderate to high levels of stress during diagnosis delivery. This finding is supported by a review of studies that used self-report and psychophysiological measures and showed that during the communication of bad news, doctors experienced moderate levels of stress, with stress reactions lasting for hours or even days after the diagnosis [32]. The experience of stress could potentially be linked to participants reporting ‘perceived lack of time’ and ‘fear of causing distress’ as the barriers they often experienced while breaking bad news and qualitative comments indicating that diagnosis delivery could sometimes be emotionally ‘*draining*’. Despite the emotional toll of breaking bad news, dealing with patients’ emotional reactions did not seem to present a particular challenge for the participants of this survey. However, studies of patients with MNDDs have shown that patients are often dissatisfied with the lack of empathy shown by doctors during diagnosis delivery [10]. The seemingly contradictory finding here in that participants in this survey reported strong competency in this domain could either be attributed to participation bias (see limitations below) or different views and expectations between patients and professionals regarding the emotional aspects of the consultation.

Finally, most participants in this study reported not following any specific strategy or guidelines when delivering an MNDD diagnosis. Although step-wise protocols for breaking bad news have been criticized for potentially focusing more on the process than the people involved, their contribution to the medical practice and their emphasis on empathy and individualised information provision is acknowledged [33]. Despite their usefulness, these protocols, such as SPIKES [11], have been developed and have mostly been used within oncology settings. In addition, when it comes to MNDDs, only EFNS [19] and NICE MND [12] guidelines adequately addressed the topic of diagnosis delivery, while, for the other conditions, guidance was mostly limited to what kind of information to impart at diagnosis and we found no guidelines for HD. This could partially explain why most participants did not follow any specific strategies when breaking bad news for MNDDs. However, most participants in the survey indicated their interest in receiving further training on breaking bad news and responding to patients’ emotions.

### Implications for research and practice

This exploratory survey highlighted several aspects of diagnosis delivery for MNDDs which could be improved. Limited consultation times and inability to offer early follow-ups were often reported by participants as factors that hampered optimal diagnostic communication. This is potentially linked to staff shortages in neurology, services constraints and the NHS in general being under strain but highlights the need for organisational changes which acknowledge the importance of diagnosis delivery consultations for MNDDs. Beyond longer consultations, there is also a need for early follow-ups so the professional can provide all the relevant information and the reassurance that patients and their families need at diagnosis and will also provide the opportunity for patients to express their emotions, prepare questions and make informed decisions regarding their care [34]. When faced with limited consultation times at diagnosis, professionals should make sure that they provide tailored information to each patient, written information about their condition, discuss their plan of care, reliable sources of information and support and ensure an early follow-up, usually with a specialist nurse. Data from our survey also showed that, despite recommendations, patients were not routinely advised to bring someone to the consultation. It would be worth exploring whether this varies among conditions and what factors influence this policy. However, we suggest that for the diagnosis of all MNDDs, patients are always given the option to be accompanied by someone. Moreover, it is recommended that, when it would not cause serious diagnostic delay, doctors should avoid delivering the diagnosis before national holidays or important events for the patient, building on the good practice reported by the majority in this survey.

Regarding professionals’ manner of delivering these diagnoses, our findings suggest that participants were reluctant to provide a sense of hope to patients with MND or HD. Despite the severe life-limiting and threatening nature of these conditions, professionals should still try to explore and enhance patients’ own concepts of hope and share information which could be deemed as positive [31], such as providing reassurance for effective symptom management and long-term support by a multi-disciplinary team. This is a topic where more research and development of training would be particularly useful in order to explore professionals’ working in neurology concepts of hope and how these affect their practice of breaking bad news. Professionals in this survey reported moderate levels of stress when communicating an MNDD diagnosis, they acknowledged the difficulty of the task and briefly discussed the emotional aspect of being the bearer of such bad news. Further qualitative research on professionals’ lived experience of communicating these diagnoses would help develop a deeper understanding on their perspectives and how they cope with giving these diagnoses on an emotional level. Exploring the opportunity for psychological input and the involvement of a multidisciplinary team in the process of breaking bad news would also be beneficial. This knowledge would be useful for developments in the design of medical education in neurology, adequately supporting professionals with this challenging task and eventually improving the patient experience. In addition, although diagnosis delivery is a critical milestone in patients’ care, future research could also address other forms of breaking bad news in MNDDs such as the initiation of discussions around advanced directives.

Finally, most participants in this study indicated an interest in receiving further training in breaking bad news and reported low familiarity with published protocols of best practice. Professionals are encouraged to familiarise themselves with such protocols and best practice guidelines for breaking bad news which could be incorporated as a part of their training. Even though the SPIKES protocol [11] was initially developed for use within oncology, some data indicate its relevance for use within neurology. In particular, MND patients were more likely to judge neurologists’ skills as ‘above average’ when they delivered the news in a way that resembled the steps described in SPIKES [35]. Nevertheless, further research incorporating both professionals’ and patients’ and families’ needs and perspectives could help develop more tailored guidelines for neurology.

### Limitations

The survey’s relatively small sample size (*N* = 49) could be considered one of the study’s limitations. However, the recent ABN’s Neurology Workforce Survey [36] identified a serious lack of UK neurologists within the UK, with the second lowest number of neurologists per head of population in Europe. It is estimated that 958 are practising in the UK [37] and although 84% of them run general neurology clinics [36], not all of them will deliver the diagnosis for the conditions included here. In addition, recruiting NHS healthcare staff in health research has been increasingly difficult due to often severe staff shortages and pressure being placed on clinicians [38]. Ultimately, this is a descriptive survey which gave the opportunity to these professionals to report on a significant aspect of their clinical practice and, through qualitative comments, discuss how it has been affected by the current NHS climate. Moreover, the results of this survey could be affected by participation bias. In particular, it is likely that most people who completed the survey were interested in the topic [39], and thus potentially better at breaking bad news and acknowledging the complexity of the task, and thus the findings may not be entirely representative of all neurology professionals.

## Conclusion

Medical professionals delivering the diagnosis of MNDDs are faced with the challenge of communicating effectively, but also sensitively, being honest, but also providing a sense of hope. This was the first survey in the UK to address neurologists’ practice and experiences in communicating these diagnoses. It was clear that for participants of this survey giving such bad news was an intrinsically challenging and stressful task which became even harder due to long waiting times for appointments in neurology and limited consultation times. Participants reported often spending a sub-optimal amount of time for these diagnostic consultations and discussed how the incurable nature of MNDDs, the uncertainty about the rate of disease progression and the, occasionally, young disease onset made such diagnostic consultations more challenging. Nevertheless, participants in this study showed signs of good practice regarding the setting of the consultation and providing appropriate and honest information at diagnosis. Apart from time restrictions and issues related to capacity, this study highlights other areas of improvement such as including patient’s family routinely in the appointments and providing some sense of hope even for conditions with a poor prognosis. Participants also reported low familiarity with breaking bad news protocols and best practice guidelines but also indicated an interest for further training in this domain.

## Supplementary Information

**Additional file 1.**

## Data Availability

The datasets used and analysed during the current study are not publicly available because participants have not given consent for public availability of their data. Data are available from the corresponding author on reasonable request.

## Abbreviations

ABN: Association of British Neurologists
ENFS: European Federation of Neurological Societies
HD: Huntington’s disease
HRA: Health Research Authority
MND: Motor neurone disease
MNDD: Motor neurodegenerative condition
MS: Multiple sclerosis
NHS: National Health Service
NICE: National Institute for Health and Care Excellence
PD: Parkinson’s disease

## Footnotes

1 In this study we focused on the diagnosis of the onset of HD, the start of the symptomatic stages of the condition, which can often be years after confirmation of gene positive status
