# Supplementary material for: Neurologists’ current practice and perspectives on communicating the diagnosis of a motor neurodegenerative condition: a UK survey
Source: BMC Neurol. 2021 Jan 22;21:34. doi: 10.1186/s12883-021-02062-6 (PMC7821644; doi:10.1186/s12883-021-02062-6)
Supplement: Supplementary file 1 — Additional file 1. [file 12883_2021_2062_MOESM1_ESM.docx]

**Survey questions**

**Sociodemographic information**

**Q2** Please indicate your age

- Under 30
- 31-40
- 41-50
- 51-60
- 61 or older

**Q1** What is your gender?

- Male
- Female
- Other

**Q3** Are you a consultant neurologist or a specialist registrar?

- Consultant neurologist
- Neurology specialist registrar
- Other (please specify) ________________________________________________

**Q4** If you are a consultant neurologist, how many years have you been practising?

____________

**Q5** In which part of the UK are you mainly currently practising? (tick all that apply)

- England
- Scotland
- Wales
- Northern Ireland

**Q6** Practice sector (select both if both apply)

- NHS
- Private practice

General questions about giving a diagnosis for motor neurodegenerative diseases

**Q7** For which of the following conditions have you given a diagnosis before? Tick all that apply

- Parkinson's disease (PD)
- Multiple sclerosis (MS)
- Huntington's disease (HD)
- Motor neurone disease (MND)

**Q8** Approximately how many diagnoses for these neurological conditions have you communicated so far?

|  | 1 - 20 | 21 - 50 | 51 - 100 | more than 100 |
| --- | --- | --- | --- | --- |
| PD |  |  |  |  |
| MS |  |  |  |  |
| HD |  |  |  |  |
| MND |  |  |  |  |

**Q9** Do you tell someone with a neurological condition their diagnosis?

|  | Always | In most cases | In some cases | Just in part | Never |
| --- | --- | --- | --- | --- | --- |
| PD |  |  |  |  |  |
| MS |  |  |  |  |  |
| HD |  |  |  |  |  |
| MND |  |  |  |  |  |

**Q10** Please state the reason(s) for your answer above

____________________________________________________________________________

|  | Months OR | Weeks |
| --- | --- | --- |
| PD |  |  |
| MS |  |  |
| HD (when genetic status was unknown) |  |  |
| MND |  |  |

**Q11** What would be the average length of time between your first clinical consultation with the patient and the delivery of the diagnosis?

**Q12** On average, how long is the consultation when you deliver a diagnosis?

|  | Minutes |
| --- | --- |
| PD |  |
| MS |  |
| HD |  |
| MND |  |

**Q13** Do you ever require more than one consultation to fully explain the diagnosis?

|  | Always | Sometimes | Never |
| --- | --- | --- | --- |
| PD |  |  |  |
| MS |  |  |  |
| HD |  |  |  |
| MND |  |  |  |

**Q14** If you do, please indicate the reasons you would require more consultations.

________________________________________________________________

**Q15** Do you ever refer patients to a different physician or service, who will then deliver the diagnosis of a neurological condition (e.g the patient's GP)

- Yes (please indicate on which occasions and for which of the conditions we are focusing on) ________________________________________________________________
- No

**Q16** Is it your service's policy to ask the patient to bring someone to the consultation?

- Always
- Most of the time
- About half the time
- Sometimes
- Never

**Q17** How often do you include a nurse or another health care professional in the consultation?

- Always
- Most of the time
- About half the time
- Sometimes
- Never

**Q18** Do you refrain from giving a diagnosis at any specific time or day?

- Yes (please specify) ________________________________________________
- No

**Q19** When you first give the diagnosis, which of these clinical aspects do you consider should be discussed with the patient? (Tick all that apply)

- How the diagnosis was reached
- The degree of certainty of the diagnosis
- The course/prognosis of the disease
- Possible causes of the disease
- Treatment options
- Current research
- Other (please specify)_______________________________________________

**Q20** How often do you deliver the diagnosis in a private space? (e.g in a consulting room)

- Always
- Most of the time
- About half the time
- Sometimes
- Never

**Q21** How often do you communicate a diagnosis without any interruptions?

- Always
- Most of the time
- About half the time
- Sometimes
- Never

**Q22** Do you maintain eye contact with the patient?

- Always
- Most of the time
- About half the time
- Sometimes
- Never

**Q23** Do you arrange to have suitable seating at the same level as the patient and without a desk or barrier?

- Always
- Most of the time
- About half the time
- Sometimes
- Never

**Q24** Do you promote a feeling of optimism when delivering a diagnosis?

|  | Definitely yes | Probably yes | Might or might not | Probably not | Definitely not |
| --- | --- | --- | --- | --- | --- |
| PD |  |  |  |  |  |
| MS |  |  |  |  |  |
| HD |  |  |  |  |  |
| MND |  |  |  |  |  |

**Q25** When talking about treatment, do you enable the patient to express their personal needs and preferences?

- Always
- Most of the time
- About half the time
- Sometimes
- Never

**Q26** Do you believe patients are given enough time to ask questions and express their emotions?

- Definitely yes
- Probably yes
- Might or might not
- Probably not
- Definitely not

**Q27** In addition to copying the patient to the standard mails that are sent to their GP, do you provide any other information tailored to their case in written form?

- Always
- Most of the time
- Occasionally
- Seldom
- Never

**Q28** How often do you share information about local support groups and national charities?

|  | Always | Most of the time | About half the time | Sometimes | Never |
| --- | --- | --- | --- | --- | --- |
| PD |  |  |  |  |  |
| MS |  |  |  |  |  |
| HD |  |  |  |  |  |
| MND |  |  |  |  |  |

**Q29** Do you initiate a follow up communication and support plan following giving the diagnosis?

- Always
- Most of the time
- Occasionally
- Seldom
- Never

**Q30** On average, how soon after giving the diagnosis would you follow up the patient?

|  | WEEKS OR | MONTHS |
| --- | --- | --- |
| PD |  |  |
| MS |  |  |
| HD |  |  |
| MND |  |  |

Experiences of breaking bad news

**Q31** Do you think delivering the diagnosis for the conditions discussed is a difficult task for the physician?

- Definitely yes
- Probably yes
- Might or might not
- Probably not
- Definitely not

**Q32** What is the most difficult part of communicating the diagnosis for these neurological conditions? (Please tick all that apply)

- Being honest but not taking away hope
- Dealing with the patient’s emotion (e.g. crying, anger)
- Spending the right amount of time
- Involving the family of the patient
- Involving patient or family in decision-making
- Other (please specify):

________________________________________________

________________________________________________

**Q33** How would you asses the stress and anxiety you experience during the delivery of a diagnosis of this nature?

- None
- Slight
- Moderate
- High
- Very high

**Q34** Research has shown that there are several potential barriers a physician might face during a breaking bad news consultation. Based on your experiences, please indicate how often, if ever, the factors listed below affect the way you deliver the diagnosis for a neurological condition.

|  | Never | Sometimes | About half the time | Most of the time | Always |
| --- | --- | --- | --- | --- | --- |
| Fear of the messenger getting blamed for bad news |  |  |  |  |  |
| Fear of causing distress |  |  |  |  |  |
| Fear of not having all the answers |  |  |  |  |  |
| Fear of being asked difficult questions |  |  |  |  |  |
| Patients/relatives being non-receptive or challenging |  |  |  |  |  |
| Lack of/insufficient training in breaking bad news |  |  |  |  |  |
| Perceived lack of time |  |  |  |  |  |
| Excessive workload |  |  |  |  |  |
| Information flaws within the service (e.g not having all patient's files/tests available) |  |  |  |  |  |

**Q35** Please outline any specific challenges when communicating the diagnosis, associated with the particular clinical nature of these conditions (e.g. lack of an effective treatment, different types of MS etc.)

|  | Challenges |
| --- | --- |
| PD |  |
| MS |  |
| HD |  |
| MND |  |

**Q36** Please rate the level of difficulty in the different aspects of delivering the diagnosis below

|  | Not at all difficult | A little bit difficult | Somewhat difficult | Difficult | Very difficult |
| --- | --- | --- | --- | --- | --- |
| Finding enough time to deliver the diagnosis |  |  |  |  |  |
| Responding to patient's emotions (crying, anger, disbelief) |  |  |  |  |  |
| Evaluate the patient's preferences about the amount and nature of information they want |  |  |  |  |  |

**Q37** In general, how would you self-assess how well you communicate the news for a motor neurodegenerative condition?

- Very good
- Good
- Fair
- Poor
- Very poor

**Q38** How confident are you that patients leave the consultation having taken in all the information relevant to them at that point?

|  | Very confident | Confident | Not sure | Not confident | Really not confident |
| --- | --- | --- | --- | --- | --- |
| PD |  |  |  |  |  |
| MS |  |  |  |  |  |
| HD |  |  |  |  |  |
| MND |  |  |  |  |  |

Q39 In general, how satisfied do you think your patients are with the way the diagnosis is delivered?

- Very satisfied
- Somewhat satisfied
- Neither satisfied nor dissatisfied
- Somewhat dissatisfied
- Very dissatisfied

Education and Training needs

**Q40** Have you had any specific education or practical training for giving a motor neurodegenerative disease diagnosis?

- No training
- Part of degree/formal education
- Clinical training post qualification
- Sat in with clinicians in breaking bad news interviews
- Other (please specify): ________________________________________________

**Q41** Have you had any training in the techniques of responding to patients’ emotions?

- No training
- Part of degree/formal education
- Clinical training post qualification
- Sat in with clinicians in breaking bad news interviews
- Other (please specify): ________________________________________________

**Q42** Do you follow any specific strategy or best practice guidelines when delivering a motor neurodegenerative disease diagnosis?

- Yes (please specify) ________________________________________________
- No

**Q43** Would you be interested in receiving further training/education in breaking bad news and techniques of responding to patients’ emotions to this news?

- Very interested
- Somewhat interested
- Not interested

**Q44** Are there any other comments you would like to make regarding the topic in general or the survey questions? 

________________________________________________________________

________________________________________________________________

________________________________________________________________
